# Traditional Chinese medicine (*Shun-Qi-Tong-Xie* Granule) for irritable bowel syndrome: study protocol for a randomised controlled trial

**DOI:** 10.1186/1745-6215-15-273

**Published:** 2014-07-07

**Authors:** Xiao-xiang Wang, Rui-jie Luo, Bin She, Yan Chen, Jia Guo

**Affiliations:** 1 Department of Integrated Traditional and Western Medicine, West China Hospital of Sichuan University, 37 Guoxue Lane, Chengdu 610041, Sichuan Province, People’s Republic of China

**Keywords:** Irritable bowel syndrome, Diarrhea, Traditional Chinese medicine, *Shun-Qi-Tong-Xie* Granule, Randomised controlled trial

## Abstract

**Background:**

Irritable bowel syndrome (IBS) is a common gastrointestinal functional disorder with no effective therapy. Traditional Chinese medicine (TCM) is one of the most common complementary therapies in China. We designed this study to evaluate the efficacy and safety of *Shun-Qi-Tong-Xie* Granule (SQTX Granule), a TCM treatment, in patients with IBS with diarrhea (IBS-D).

**Methods/Design:**

A randomised, double-blinded, placebo-controlled, multi-centre, superiority clinical trial to evaluate the efficacy and safety of SQTX Granule is proposed. Eligible patients (Rome III) with IBD-S will be randomly assigned into SQTX Granule group and the placebo group. Patients will receive a 28-day treatment and a 2-month follow-up. The primary outcome measures include the scores of IBS-quality of life (IBS-QOL) rating scale and IBS-symptom severity scale (IBS-SSS) rating scale. The secondary outcome measures include the improvement of symptom scores, and the duration of abdominal pain and diarrhea.

**Discussion:**

According to TCM theory, SQTX Granule has a regulating effect on abdominal pain, diarrhea and the syndrome of liver-spleen disharmony, which is similar to the symptoms of IBS-D. This study will provide objective evidence to evaluate the efficiency and safety of SQTX Granule in IBS-D treatment.

**Trial registration:**

ChiCTR-TRC-14004241.

Date of registration: 9 February 2014.

## Background

IBS refers to a clinical syndrome that presents as a common chronic gastrointestinal disorder. Patients suffer from abdominal pain or discomfort, bloating, and altered bowel habit, which vary in both degree and frequency, from slight to severe and from recurrent to persistent, respectively [1]. The disease is one of the most common functional gastrointestinal (GI) disorders with a reported prevalence of approximately 5 to 20% worldwide [1-3]. IBS is more commonly diagnosed in women than in men, and in patients younger than 50 years [1,4,5]. IBS considerably reduces patients’ quality of life, interfering with their working ability and social activity, and it also results in a significant economic burden through decreased work productivity and increased healthcare costs [6]. With the accelerated pace of life and changes in diet in recent years, neurological, psychiatric and infectious factors have caused an upward trend in the incidence of IBS [7-9]. No biomarkers have been found for IBS diagnosis [10], so IBS is diagnosed by assessing symptoms, according to the Rome III criteria [11,12]. According to these criteria, four subtypes of IBS were recognised: IBS with constipation (IBS-C), IBS-D, mixed IBS (IBS-M), and unsubtyped IBS (IBS-U). Each IBS patient could switch from one subgroup to another over time. Current medication approaches for IBS are based on symptom reduction, yet many patients remain undertreated; meanwhile, many medication approaches are associated with side effects that result in less benefit to the patient, particularly in treatment for long-term relief of abdominal pain or discomfort.

Due to unsatisfactory treatment effectiveness, the search for and use of complementary and alternative medicines (CAM) has increased during the past several years around the world [13-15]. Studies indicate an improvement both in the abdominal complaints and the quality of life in IBS patients adopting CAM treatment, which includes dietary supplements, TCM, acupuncture, moxibustion and botanicals [13-16]. TCM is one of the most common CAMs used in China, and it has an over 5,000-year history of applications for various diseases, including IBS-D [17]. According to TCM theory, one of the most important principles of diagnosis and treatment is symptom differentiation, which is based on four diagnostic methods (inspection, listening and smelling, inquiry and palpation) to collect clinical manifestations, including symptoms and TCM signs.

In IBS-D, the most common symptom type is liver-spleen disharmony, and it is primarily characterised by abdominal pain/discomfort, diarrhea, chest/flank/abdominal distension, belching, frequent sighing and emotion-related symptoms. According to the basic principles of TCM, IBS-D with liver-spleen disharmony is treated by relieving stagnation of liver *qi*, as well as improving the transportation function of the spleen. First recorded in the TCM classic ‘*Jingyue's Complete Works*’, *Tong-Xie-Yao-Fang* (TXYF) decoction has been applied since the Ming Dynasty (16^th^ century) to cure abdominal pain with diarrhea (liver-spleen disharmony). Modified from TXYF decoction, *Shun-Qi-Tong-Xie* (SQTX) Granule treats abdominal pain, distention and diarrhea in patients suffering from IBS-D (liver-spleen disharmony), and it is formulated with the Chinese herbs listed in Table 1, which have all been approved by the China Food and Drug Administration (CFDA) [18]. In preclinical trials and toxicological studies, no evidence of toxic effects has been found with SQTX Granule. Pharmacologic experiments showed that it has the effects of inducing spasmolysis, analgesia and reducing diarrhea in rats with diarrhea caused by stress. Phase II clinical trials also showed that SQTX Granule can improve symptoms of abdominal pain/discomfort and the degree and frequency of diarrhea for patients with IBS-D (liver-spleen disharmony).

**Table 1:** **Standard formulation of *Shun-Qi-Tong-Xie *** **(SQTX) Granule**

**Pinyin name**	**Latin name**
*Baishao*	*Paeonia lactiflora Pall.*
*Baizhu*	*Atractylodes macrocephala Koidz*.
*Xiebai*	*Allium macrostemon Bunge*.
*Cheqianzi*	*Hegba Plantaginis Asiaticae*
*Foshou*	*Citrus medica L. var. sarcodactylis Swingle*
*Boheyou*	*Oleum Mentha haplocalyx* Briq.

Chinese herbs have been used for a long time with good effects and few adverse events; however, a randomised, double-blind, placebo-controlled trial is still necessary to prove the efficacy and safety of any medication. As a phase III clinical trial, the objective of this study is to evaluate the efficacy and safety of SQTX Granule with a placebo in patients with IBS-D (liver-spleen disharmony). The results of this study will provide evidence regarding the value of SQTX Granule as an intervention for IBS-D.

## Methods/design

### Design

**Figure 1:**
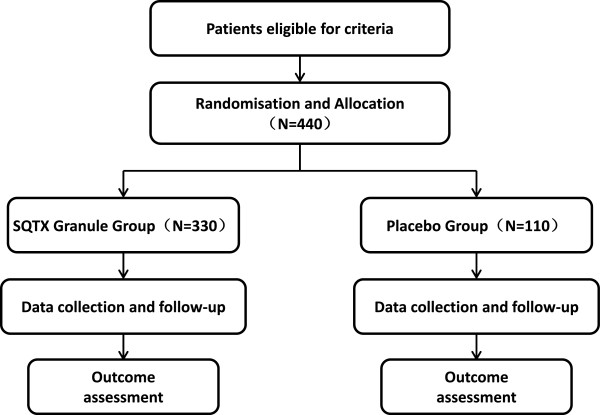
**Study flow chart.** The flow chart of enrolment, allocation, intervention and assessment.

This study is designed as a randomised, double-blinded, placebo-controlled, multi-centre, superiority clinical trial. This study is a phase III clinical trial and has been authorised by the CFDA (Approval Number 2007 L02218). In addition, the study is registered with the Chinese Clinical Trial Registry (ChiCTR-TRC-14004241). The study is financially supported by Shaanxi Bosen Biological Pharmaceutical Group Co. Ltd, Xi’an, China. This funding source is providing test drugs, but it had no role in the design of this study and does not have any responsibility for analyses, interpretation of the data or the decision to submit results. Trained research nurses introduce the trial to patients, and give them information sheets and consent forms. All patients have to give their written informed consent prior to enrolment. The study’s flow chart is shown in Figure 1.

### Ethics

The trial protocol is conducted in accordance with the Good Clinical Practice Guidelines and the Declaration of Helsinki (2008) [19]. Ethics approval has been obtained from the Clinical Trials and Biomedical Ethics Committee of West China Hospital of Sichuan University (Number TCM-2013-01). Written informed consent will be obtained from each patient.

### Recruitment

A total of 440 Chinese patients who fulfill the screening criteria will be recruited at eight large comprehensive hospitals in China: 1) West China Hospital of Sichuan University, 2) Affiliated Hospital of Chengdu University of TCM, 3) The First Affiliated Hospital of Heilongjiang University Of Chinese Medicine, 4) Affiliated Hospital of Jiangxi University of TCM, 5) Shanxi Provincial Hospital of TCM, 6) The First Affiliated Hospital of Guangxi University of Chinese Medicine, 7) Gansu Provincial Hospital of TCM and 8) Affiliated Hospital of Changchun University of Chinese Medicine. Each centre will recruit 55 patients through posters in local hospitals.

### Sample size calculation

According to the results of Phase II clinical trial, the response rates of SQTX Granule and placebo were 0.51 and 0.33, respectively. Assuming the response rates for TCM and placebo were 0.5 and 0.35, the sample size was calculated under the setting of alpha = 0.05 and beta = 0.80. Taking into account a drop-out of 15%, the sample size needs to be 150 cases for each group. A total of 440 patients will be recruited in this trial, including 330 cases for the SQTX Granule group and 110 cases for the placebo group in a 3:1 ratio.

### Randomisation

With a stratified block randomisation method, a 440-case randomisation arrangement was carried out by an independent statistician according to the sequence generated with SAS software; this lists a serial number from 001 to 440 corresponding to consecutive allocation (with a randomisation scheme). Each centre receives consecutively coded drugs. All of the drugs provided by the pharmaceutical company will be numbered with a label according to the randomisation schedule.

### Blinding

This trial is a double-blind trial. The first level is for the case number corresponding to groups (group A, group B), and the second level is for the group corresponding to intervention (the test and placebo groups). The numbers are kept in opaque sealed envelopes. The double levels of blinding are sealed separately, and given to the leader and the sponsor of the clinical research. Emergency letters are sent to each of the centres, saved with the test drug, and properly preserved until the end of the trial. Treatment assignments will not be revealed and are blinded to the patients and investigators (including statisticians) until the entire study is completed. Code-breaking should only occur with the permission of the research centre leader in exceptional circumstances when serious adverse events happen and knowledge of the actual treatment is absolutely essential for further management of the patient, and a report should be submitted to the leader of the trial within 24 hours. For the first level of unblinding, before statistical analysis, the blind code will be disclosed to group A and group B. For the second level of unblinding, group A and group B will be disclosed to the test placebo groups in a summary meeting.

### Western medicine diagnostic criteria for IBS-D

Diagnosis of IBS-D is established according to the Rome III criteria [11,12], mode listed in Table 2.

**Table 2:** Mode of irritable bowel syndrome (IBS)

**Question**	**Score**
A) In the last 3 months, how often did you have hard or lumpy stools?	0 Never or rarely
	1 Sometimes
	2 Often
	3 Most of the time
	4 Always
B) In the last 3 months, how often did you have loose, mushy or watery stools?	0 Never or rarely
	1 Sometimes
	2 Often
	3 Most of the time
	4 Always
**Mode**	**Definition**
IBS-C	A > 0 and B = 0
IBS-D	A = 0 and B > 0
IBS-M	A > 0 and B > 0
IBS-U	A = 0 and B = 0

Diagnostic criteria*:

Recurrent abdominal pain or discomfort** at least three days/month in the last three months associated with two or more of the following:

1. Improvement with defecation

2. Onset associated with a change in frequency of stool

3. Onset associated with a change in form (appearance) of stool

*Criteria fulfilled for the last three months with symptom onset at least three months prior to diagnosis

**‘Discomfort’ means an uncomfortable sensation not described as pain.

In pathophysiology research and clinical trials, a pain/discomfort frequency of at least two days a week during the screening evaluation is recommended for subject eligibility.

### Diagnostic criteria for TCM syndrome differentiation

The TCM diagnosis of liver-spleen disharmony and diarrhea syndrome is based on the Guidelines for Clinical Research of New Chinese Medicine [20].

For diagnosis with liver-spleen disharmony and diarrhea syndrome, patients will have both of the primary and at least two of the secondary symptoms listed in Table 3, as well as the TCM signs for the tongue and pulse.

**Table 3:** Traditional Chinese medicine (TCM) diagnostic criteria

**Category**	**Symptoms and signs**
Primary symptoms	1) Abdominal pain or discomfort, 2) Diarrhea
Secondary symptoms	1) Chest/flank or abdominal distension, 2) Diarrhea aggravation related to emotional tension, depression or anger, 3) Belching, 4) Decreased appetite, 5) Frequent sighing, 6) Fatigue
TCM signs for the tongue	Pink tongue, white/thick/greasy tongue coating
TCM signs for the pulse	Wiry pulse

### Inclusion criteria

• Diagnosis of IBS-D according to Western medicine

• Diagnosis of liver-spleen disharmony and diarrhea syndrome according to TCM

• Aged between 18 and 65 years old

• Able to understand and sign a written informed consent

### Exclusion criteria

• Diagnosis of IBS-C, IBS-M or IBS-U according to Western medicine

• A history of previous abdominal surgery

• Other organic diseases causing abdominal pain and diarrhea

• History of usage of other medication for IBS-D during the last week

• Patients taking any medication for other diseases which would impact the trial

• Pregnant and lactating women

• Patients who have an allergic constitution or are allergic to the trial medicine

• Patients with serious primary diseases of the heart, liver, kidney, blood system or endocrine system, and patients with tumours or AIDS

•Mentally or legally disabled patients

### Other criteria

Withdrawal of participants:

• Patients are determined as requiring withdrawal based on disease progression, allergic reactions, serious adverse events, or poor efficacy

• Serious complications

• Unblinding individuals for any reasons

• Voluntarily quitting

• Unable to complete the scheduled follow-up

Reasons for withdrawing patients would be noted in Case Report Forms (CRFs), and the last data would be included in data analysis.

Note: 1) Patients cured in a course of treatment are not regarded as cases for withdrawal.

2) Patients withdrawn due to poor efficacy after a half course of treatment (14 days) are regarded as not recovered but not withdrawn, and they would be included in the Full Analysis Set (FAS) and the Per Protocol Set (PPS).

### Dropout of participants

• Misdiagnosis

• Using forbidden drugs or treatments in the course of the trial

• Taking no medication during the trial

• No evaluable records after medication

### The whole study will be terminated if any of the following occurs

• Poor curative effect is found during the study

• Significant deviation from the protocol

• Flawed protocol

• The sponsors decide to terminate the trial due to management or funding problems

• At the decision of the pharmaceutical supervisory and administrative department

### Concomitant treatments and forbidden drugs

Usage of any other Western medication or Chinese medicines that have similar effects to SQTX Granule, or may affect the analysis of the final results, is strictly prohibited. Medications used to control other conditions of the participants, such as hypertensive or diabetic medications, are allowed. The dosage, duration and name of any concomitant treatment or medication must be recorded carefully in the CRFs.

### Test drugs

The test drugs are SQTX Granule and placebo, which are indistinguishable from the actual medication. The placebo has the same shape, size, taste, colour, package and Lot number.

### Intervention

**Figure 2:**
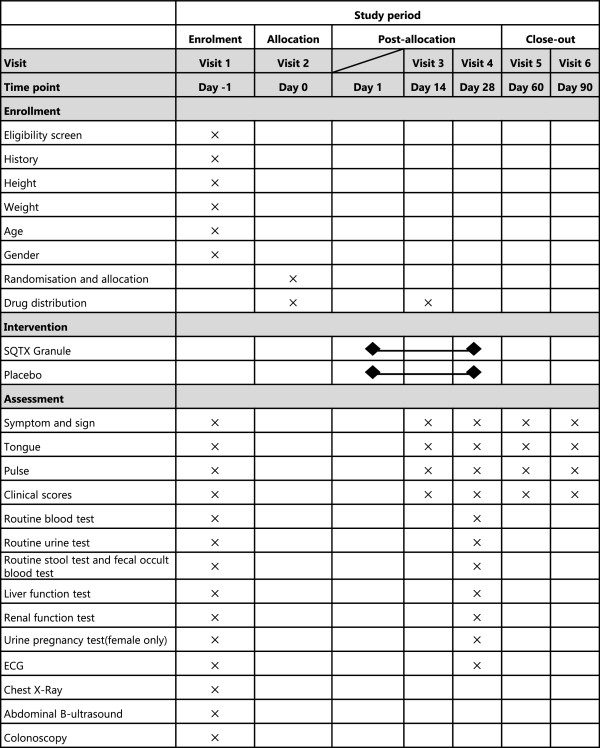
**Study schedule for patients.** After the enrolment and allocation, participants will receive the administration for four weeks and a follow-up of eight weeks. The time-points of assessment are shown in the schedule.

Patients are administered SQTX Granule or placebo in one bag (0.5 g per bag) dissolved in warm water three times daily for 28 days. The follow-up visits last for one week after the treatment. The measurements that need to be performed at each visit are listed in Figure 2.

### Outcome assessment

#### Primary outcome

• Scores of IBS-quality of life rating scale (IBS-QOL) rating scale [21]

• Scores of IBS-symptom severity scale (IBS-SSS) rating scale [22]

### Secondary outcome

**Figure 3:**
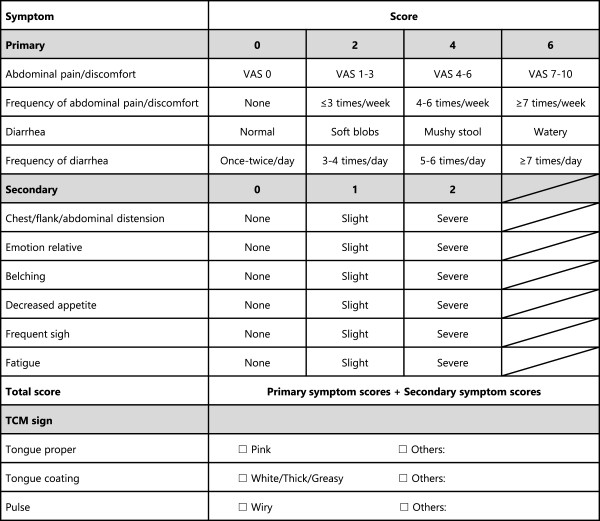
**Symptom score system.** As a secondary outcome, a Traditional Chinese medicine (TCM) symptom score system is introduced into this protocol.

• The symptom score system is introduced to quantify the degree of abdominal pain, diarrhea, stool consistency and other concomitant symptoms as shown in Figure 3, following the Guidelines for Clinical Research of New Chinese Medicine [20].

• The treatment efficacy evaluation system is introduced to evaluate the improvement of symptoms using the percentage of symptom score reduction (PSSR). The PSSR for a patient after treatment is calculated according to the following formula:

$$\begin{array}{ll} \;\text{PSSR} & =\left(\frac{\text{Symptom}\;\text{score}\;\text{at}\;\text{Day}\;0‒\text{Symptom}\;\text{score}\;\text{at}\;\text{Day}\;28}{\text{Symptom}\;\text{score}\;\text{at}\;\text{Day}\;0}\right)\\ & \;\times 100\% \end{array}$$

Full recovery: PSSR ≥ 90%, Good recovery: 90% > PSSR ≥ 70%, Modest recovery: 70% > PSSR ≥ 30%, No recovery: PSSR < 30%.

• Decreasing and disappearing time of abdominal pain and diarrhea:

  Decreasing time = (The day pain/diarrhea decrease) - Day 1

  Disappearing time = (The day pain/diarrhea disappear) - Day 1

### Safety outcome

A routine blood test, routine urine test, routine stool test, faecal occult blood test, urine pregnancy test (women only), liver function test, renal function test and an electrocardiogram are administered for safety outcomes, which are monitored both before and after treatment.

### Data management

After review by clinical inspectors, completed CRFs will be sent to a specified statistics centre, and data entry and management is completed by two individual data administrators to ensure the accuracy of the data. To ensure the database is created correctly after blind review, data will be locked by the clinical research leader, the sponsor and the statistical analyst, so that no changes are allowed to the locked data.

### Adverse events

Adverse events will be recorded and graded in detail throughout the study. When a severe adverse event occurs, participants will be provided with every necessary treatment, and the event must be reported to the leader of the trial, ethics committees, sponsors and CFDA within 24 hours.

### Data analysis

Statistical analysis will be conducted based on an intention-to-treat (ITT) principle. All subjects who were treated with at least one dose of the study drug and had at least one clinical observation after starting the treatment comprise FAS. All treated subjects without any major protocol deviations comprise PPS. The statistical analysis will be performed using the SAS 8.1 software (SAS, Cary, NC, USA). The statistical significance is defined as a two-sided *P*-value of < 0.05. Baseline differences among the groups will be assessed with the use of the one-factor analysis of variance or the Mann-Whitney *U-*test for measurement data and the χ^2^ test or the Wilcoxon test for enumeration data. For categorical variables, a χ^2^ test or Fisher’s exact test will be used. The changes from baseline to endpoint of treatment in scores will be assessed with the use of a paired *t*-test for measurement data and a signed rank test for enumeration data. Comparisons between groups will be conducted by using an analysis of variance (ANOVA) and a rank test.

## Discussion

Characterised by continuous or intermittent abdominal pain, bloating, changes of bowel habits or stool consistency, IBS is a disease lacking any changes of gastrointestinal structure and biochemical abnormalities. Moreover, greatly impacting the function of the gut, psychological stress plays a fairly important role in inducing, aggravating and perpetuating the symptoms of IBS; thus, a portion of IBS patients exhibit mental disorders, mainly anxiety and depression [23-26]. No evidence of any pathological change is found. Current Western medicine provides limited symptomatic relief to IBS patients through pharmacological treatment; however, TCM has a long history spanning thousands of years, dealing with abdominal pain and diarrhea. In recent years, a group of studies confirmed the efficiency of TCM treatment on IBS [27-29]. As the most common type of IBS, IBS-D is characterised by abdominal pain/discomfort and diarrhea, mainly accompanied with emotional instability, which is the exact indication of SQTX Granule in our study. Pharmacological and toxicological research with SQTX Granule demonstrated the efficacy and safety in preclinical trials, respectively, and phase II clinical trials also indicate the improvement of symptoms without significant adverse events. A randomised, multi-centre, double-blind, placebo-controlled, superiority, phase III clinical trial will be helpful for further clarification of these results.

## Trial status

At the time of manuscript submission, patient recruitment for the trial is on-going.

## Abbreviations

ANOVA: analysis of variance; CAM: complementary and alternative medicines; CFDA: China Food and Drug Administration; CRFs: Case Report Forms; FAS: Full Analysis Set; GI: gastrointestinal; IBS: irritable bowel syndrome; IBS-C: IBS with constipation; IBS-D: IBS with diarrhea; IBS-M: mixed IBS; IBS-QOL: IBS-quality of life; IBS-SSS: IBS-symptom severity scale; IBS-U: unsubtyped IBS; ITT: intention-to-treat; PPS: Per Protocol Set; PSSR: percentage of symptom score reduction; SQTX Granule: *Shun-Qi-Tong-Xie* Granule; TCM: Traditional Chinese medicine; TXYF: *Tong-Xie-Yao-Fang*; VAS: Visual Analogue Scale.

## Competing interests

The authors declare that they have no competing interests.

## Authors’ contributions

XXW participated in the design of the study and drafted the manuscript. RJL participated in the statistical design and helped in the design of the study. BS and YC helped to draft the manuscript. JG was the general supervisor for this research and participated in the critical revision of the manuscript. All authors read and approved the final manuscript.

## References

[B1] Agréus L Svärdsudd K Nyrén O Tibblin G Irritable bowel syndrome and dyspepsia in the general population: overlap and lack of stability over time Gastroenterology 1995 109 671 680 7657095 10.1016/0016-5085(95)90373-9

[B2] Brandt LJ Chey WD Foxx-Orenstein AE Schiller LR Schoenfeld PS Spiegel BM Talley NJ Quigley EM An evidence-based position statement on the management of irritable bowel syndrome Am J Gastroenterol 2009 104 Supp 1 S1 S35 19521341 10.1038/ajg.2008.122

[B3] Rey E Talley NJ Irritable bowel syndrome: novel views on the epidemiology and potential risk factors Dig Liver Dis 2009 41 772 780 19665952 10.1016/j.dld.2009.07.005

[B4] Talley NJ Zinsmeister AR Melton LJ 3rd Irritable bowel syndrome in a community: symptom subgroups, risk factors, and health care utilization Am J Epidemiol 1995 142 76 83 7785677 10.1093/oxfordjournals.aje.a117548

[B5] Thompson WG Irvine EJ Pare P Ferrazzi S Rance L Functional gastrointestinal disorders in Canada: first population-based survey using Rome II criteria with suggestions for improving the questionnaire Dig Dis Sci 2002 47 225 235 11837727 10.1023/a:1013208713670

[B6] McFarland LV State-of-the-art of irritable bowel syndrome and inflammatory bowel disease research in 2008 World J Gastroenterol 2008 14 2625 2629 18461647 10.3748/wjg.14.2625 PMC2709056

[B7] Labus JS Hubbard CS Bueller J Ebrat B Tillisch K Chen M Stains J Dukes GE Kelleher DL Naliboff BD Fanselow M Mayer EA Impaired emotional learning and involvement of the corticotropin-releasing factor signaling system in patients with irritable bowel syndrome Gastroenterology 2013 145 1253 1261 23954313 10.1053/j.gastro.2013.08.016 PMC4069031

[B8] Arebi N Gurmany S Bullas D Hobson A Stagg A Kamm M Review article: the psychoneuroimmunology of irritable bowel syndrome - an exploration of interactions between psychological, neurological and immunological observations Aliment Pharmacol Ther 2008 28 830 840 18637004 10.1111/j.1365-2036.2008.03801.x

[B9] Elsenbruch S Abdominal pain in irritable bowel syndrome: a review of putative psychological, neural and neuro-immune mechanisms Brain Behav Immun 2011 25 386 394 21094682 10.1016/j.bbi.2010.11.010

[B10] Barbara G Stanghellini V Review biomarkers in IBS: when will they replace symptoms for diagnosis and management? Gut 2009 58 1571 1575 19923339 10.1136/gut.2008.169672

[B11] Drossman DA The functional gastrointestinal disorders and the Rome III process Gastroenterology 2006 130 1377 1390 16678553 10.1053/j.gastro.2006.03.008

[B12] Longstreth GF Thompson WG Chey WD Houghton LA Mearin F Spiller RC Functional bowel disorders Gastroenterology 2006 130 1480 1491 16678561 10.1053/j.gastro.2005.11.061

[B13] Grundmann O Yoon SL Complementary and alternative medicines in irritable bowel syndrome: An integrative view World J Gastroenterol 2014 20 346 362 24574705 10.3748/wjg.v20.i2.346 PMC3923011

[B14] Magge SS Wolf JL Complementary and alternative medicine and mind-body therapies for treatment of irritable bowel syndrome in women Womens Health (Lond Engl) 2013 9 557 567 24161308 10.2217/whe.13.57

[B15] Dorn SD Kaptchuk TJ Park JB Nguyen LT Canenguez K Nam BH Woods KB Conboy LA Stason WB Lembo AJ A meta-analysis of the placebo response in complementary and alternative medicine trials of irritable bowel syndrome Neurogastroenterol Motil 2007 19 630 637 17640177 10.1111/j.1365-2982.2007.00937.x

[B16] Rahimi R Abdollahi M Herbal medicines for the management of irritable bowel syndrome: a comprehensive review World J Gastroenterol 2012 18 589 600 22363129 10.3748/wjg.v18.i7.589 PMC3281215

[B17] Li Q Yang GY Liu JP Syndrome differentiation in Chinese herbal medicine for irritable bowel syndrome: a literature review of randomized trials Evid Based Complement Alternat Med 2013 2013 232147 23554827 10.1155/2013/232147 PMC3608279

[B18] Commission SP Pharmacopoeia of the People's Republic of China 2010 9 Beijing: China Medical Science Press

[B19] Vijayananthan A Nawawi O The importance of Good Clinical Practice guidelines and its role in clinical trials Biomed Imaging Interv J 2008 4 e5 21614316 10.2349/biij.4.1.e5 PMC3097692

[B20] Zhen XY Guidelines for Clinical Research of New Chinese Medicine 2002 Beijing: China Medical Science Press

[B21] Patrick DL Drossman DA Frederick IO DiCesare J Puder KL Quality of life in persons with irritable bowel syndrome: development and validation of a new measure Dig Dis Sci 1998 43 400 411 9512138 10.1023/a:1018831127942

[B22] Francis CY Morris J Whorwell PJ The irritable bowel severity scoring system: a simple method of monitoring irritable bowel syndrome and its progress Aliment Pharmacol Ther 1997 11 395 402 9146781 10.1046/j.1365-2036.1997.142318000.x

[B23] Lackner JM Jaccard J Krasner SS Katz LA Gudleski GD Blanchard EB How does cognitive behavior therapy for irritable bowel syndrome work? A mediational analysis of a randomized clinical trial Gastroenterology 2007 133 433 444 17681164 10.1053/j.gastro.2007.05.014 PMC2645996

[B24] Gulewitsch MD Enck P Schwille-Kiuntke J Weimer K Schlarb AA Mental strain and chronic stress among university students with symptoms of irritable bowel syndrome Gastroenterol Res Pract 2013 2013 206574 23843782 10.1155/2013/206574 PMC3697310

[B25] Drossman DA Chang L Bellamy N Gallo-Torres HE Lembo A Mearin F Norton NJ Whorwell P Severity in irritable bowel syndrome: a Rome Foundation Working Team report Am J Gastroenterol 2011 106 1749 1760 21747417 10.1038/ajg.2011.201

[B26] Jerndal P Ringström G Agerforz P Karpefors M Akkermans LM Bayati A Simrén M Gastrointestinal-specific anxiety: an important factor for severity of GI symptoms and quality of life in IBS Neurogastroenterol Motil 2010 22 646 e179 20367800 10.1111/j.1365-2982.2010.01493.x

[B27] Bensoussan A Talley NJ Hing M Menzies R Guo A Ngu M Treatment of irritable bowel syndrome with Chinese herbal medicine: a randomized controlled trial JAMA 1998 280 1585 1589 9820260 10.1001/jama.280.18.1585

[B28] Ma YX Liu X Liu CZ Wang LP Guo G Du DQ Wang ZL Ma H Qi P Li ZF Guo YP Yi HQ Gao SZ Randomized clinical trial: the clinical effects of herb-partitioned moxibustion in patients with diarrhoea-predominant irritable bowel syndrome Evid Based Complement Alternat Med 2013 2013 605460 24454500 10.1155/2013/605460 PMC3880695

[B29] Merat S Khalili S Mostajabi P Ghorbani A Ansari R Malekzadeh R The effect of enteric-coated, delayed-release peppermint oil on irritable bowel syndrome Dig Dis Sci 2010 55 1385 1390 19507027 10.1007/s10620-009-0854-9

